# Health-Related Quality of Life and Mental Health of Children with Embryonal Abdominal Tumors

**DOI:** 10.3390/children10101720

**Published:** 2023-10-23

**Authors:** Paulina Behrendt, Michael Boettcher, Kira Tabea Zierke, Safiullah Najem, Holger Zapf, Konrad Reinshagen, Wilhelm Wößmann, Johannes Boettcher

**Affiliations:** 1 Department of Pediatric Surgery, University Medical Center Hamburg-Eppendorf, Martinistrasse 52, 20246 Hamburg, Germanymichael.boettcher@medma.uni-heidelberg.de (M.B.); safiullah.najem@uke.de (S.N.); konrad.reinshagen@uke.de (K.R.); 2 Department of Pediatric Surgery, University Medical Center Mannheim, University Heidelberg, Theodor-Kutzner-Ufer 1-3, 68167 Mannheim, Germany; 3 Department of Pediatric Hematology and Oncology, University Medical Center Hamburg-Eppendorf, Martinistrasse 52, 20246 Hamburg, Germanywilhelm.woessmann@uke.de (W.W.); 4 Department of Child and Adolescent Psychiatry, Psychosomatics and Psychotherapy, University Medical Center Hamburg-Eppendorf, Martinistrasse 52, 20246 Hamburg, Germany

**Keywords:** embryonal abdominal tumors, health-related quality of life, mental health, rare diseases, pediatric surgery

## Abstract

(1) Background: Embryonal abdominal tumors are one of the most common entities of solid childhood cancer. The present study investigates the Health-Related Quality of Life (HRQoL) and the mental health of children to obtain a comprehensive picture of their health status and uncover a possible gap in healthcare. (2) Methods: The sample consisted of 54 children who were treated for embryonal abdominal tumors and a control group of 46 children who received uncomplicated outpatient surgery. The HRQoL and the mental health were assessed by the parent proxy reports of the questionnaires Pediatric Quality of Life Inventory (PedsQL) and Strengths and Difficulties Questionnaire (SDQ). (3) Results: Children with embryonal abdominal tumors showed significantly lower HRQoL and mental health values compared to the norm data. The index group showed lower values in the social subscales of HRQoL and mental health compared to the control group. (4) Conclusions: Embryonal abdominal tumors affect the well-being of children. There is still a gap in healthcare due to children’s HRQoL and mental health, especially regarding social development. It is essential to further advance the psychological care of children and improve their chances to develop social relationships.

## 1. Introduction

Children with embryonal abdominal tumors represent an often overlooked population due to the rarity of this disease within the healthcare system [1]. All affected children and their families face significant challenges regarding disease management [2]. Previous studies have shown that the most common symptoms of childhood cancer, such as pain and sadness, may impact the well-being and the functional status of children and adolescents [3]. This highlights the importance of exploring the general Health-Related Quality of Life (HRQoL) and the mental health of this population to obtain a comprehensive picture of the health status of those children.

Embryonal abdominal tumors, as a group of rare diseases, are a group of heterogeneous cancers that result from the degeneration of immature cells usually found in the fetus [4]. Rare diseases are defined by a prevalence of less than 1:2000 [5]. The prevalence of embryonal abdominal tumors is estimated to be 1:12,500 [6], with neuroblastoma, nephroblastoma, hepatoblastoma, and rhabdoid tumors being the most common entities in the abdominal and thoracal region [7,8]. Although these tumors differ in their specific diagnosis, treatment, and prognosis, the primary therapeutic concept for all four entities is based on the oncologic trial consisting of surgery, radiation, and chemotherapy [9]. Recently, immune therapy has been evaluated in high-risk patients [10]. In addition to physical and psychological impairments during treatment, the long-term sequelae of the therapy may be very stressful [11]. In particular, late effects of treatment, such as renal insufficiency, tissue hypoplasia, and bone deformities [12], lead to lifelong limitations in daily life and represent a high level of suffering. Survival rates have improved from 20 to 80–90 percent in recent decades [13,14,15] due to excellent research in diagnostics [16,17] and therapy [18,19]. Nevertheless, it is important to survey the HRQoL and mental health of affected persons to explore a possible gap in healthcare.

The construct HRQoL can be defined as “how well a person functions in their life and his or her perceived well-being in physical, mental, and social domains of health” [20]. Mental health can be defined as the “flexibility and ability to cope with adverse life events and function in social roles” [21]. Considering embryonal abdominal tumors in light of the diathesis–stress model, the individual vulnerability characteristics associated with rare diseases can be invoked as a possible cause of decreased HRQoL and mental health [22]. According to this model, mental disorders occur when an individual’s stress tolerance level is exceeded. The term stress is considered in this context [23]. This limit is dynamic and can be influenced by certain protective or risk factors [24].

In the last decade, many studies have shown that children with rare diseases have a significantly lower HQRoL and a significantly higher risk of emotional and behavioral disorders [25,26,27,28,29,30,31]. Nevertheless, current studies do not provide a comprehensive picture of the mental health and HRQoL of children with embryonal abdominal tumors. 

Therefore, the following research questions are addressed in this paper: (1) Are there differences in the distribution of HRQoL and mental health between children with embryonal abdominal tumors and a control group in surgical treatment? (2) In which specific subscales does the study population differ from the control group? (3) How do disease-unspecific factors influence HRQoL and the mental health of children with embryonal abdominal tumors?

We expect a generally decreased HRQoL and a decreased mental health of the affected children compared to the norm data. Moreover, we expect that children with embryonal abdominal tumors would show lower values of HRQoL as well as mental health, mainly due to their physical functionality. In addition, we expect that children with levels of care, older age, and of the female gender would be more affected.

## 2. Materials and Methods

### 2.1. Study Design

In this quantitative cross-sectional observational study, two groups were investigated using standardized psychometric questionnaires between March 2022 and March 2023. The index group was defined as a patient collective treated for an embryonal abdominal tumor at the University Medical Center Hamburg-Eppendorf. In addition, a control group was surveyed that included children and their families who underwent an outpatient surgical procedure without a resulting chronic condition, congenital chronic disease, or any other cancer disease. The study design was based on a previous study conducted from April 2020 to April 2021 at the University Medical Center Hamburg-Eppendorf, which investigated the psychosocial situation of children with rare surgical diseases during the COVID-19 pandemic [30]. The study received ethical approval from the Hamburg Medical Association (2021–100605-BO-ff) and was pre-registered at ClinicalTrials.gov (NCT05245123).

### 2.2. Variables and Instruments

The primary outcomes were the HRQoL as well as the mental health of the children. The following instruments were used to assess the children’s HRQoL and mental health:

Children’s Health-Related Quality of Life: To assess HRQoL in children and adolescents between 2 and 18 years old, the Pediatric Quality of Life Inventory (PedsQL 4.0) has been shown to be a valid survey instrument [32]. This study uses a short form with a total of 15 items. The questionnaire is divided into four core scales and includes (1) physical functionality (five items), (2) emotional functionality (four items), (3) social functionality (three items), and (4) school functionality (three items). The score obtained is then converted into a standardized 0 to 100 scale. A high score indicates a good QoL. In this study, we used the parent proxy form. The German version of the instrument showed good psychometric properties and includes normative data of healthy children and adolescents [33].

Children’s mental health: The German version of the Strength and Difficult Questionnaire (SDQ) measures the psychological adjustment of children and adolescents using five scales with five items each [34]. The 25 attributes can be negative or positive and are rated by parents using a three-point scale. Each scale contains points for (1) emotional symptoms, (2) conduct problems, (3) hyperactivity, (4) peer problems, and (5) prosocial behavior. The first four scores are then summed up to a total score. A higher score indicates greater problems in all areas except prosocial behavior. Conversely, a lower value indicates worse social competence. The questionnaire showed good psychometric properties and includes normative data of healthy children and adolescents [35].

### 2.3. Participants

Index group: The inclusion criteria for the index group were as follows: (1) all families with children between 2 and 17 years of age and (2) being treated for an abdominal embryonal tumor at the University Medical Center Hamburg-Eppendorf (3) as of 2012. To answer the questionnaire, a sufficient understanding of the German language was required. Severe acute physical, psychological, or cognitive impairments of the child constituted exclusion criteria because questionnaire collection seemed impossible or unreasonable.

The patients’ parents provided a signed informed consent. Participants were allowed to withdraw from the study at any given time. All diagnoses were reviewed by the medical personnel before inclusion in the study. In total, 90 children and adolescents treated for embryonal abdominal tumors at the University Medical Center Hamburg-Eppendorf since 2012 were identified. The response rate for the index group was 60%. Parent ratings were provided for 54 children regarding their child’s quality of life and mental health. Among these, 15 parent ratings were provided by both parents, 33 only by the mother, and 4 only by the father.

Control group: Inclusion criteria for the control group were as follows: (1) all families with children of 2–17 years of age, (2) who had an outpatient surgical procedure, and (3) without a resulting chronic condition, congenital chronic disease, or any other cancer disease. Examples include inguinal hernia surgery or testicular retention. A prerequisite for answering the questionnaire was a sufficient understanding of German. Children with a congenital or chronic oncological disease were excluded. Of 99 children and parents asked for participation, the response rate was 46.46% for the control group. Parental assessment of HRQoL and mental health was provided to 46 children. Among these, 20 parent ratings were provided by both parents, 21 only by the mother, and 4 only by the father. 

### 2.4. Statistics

Means and standard deviations were calculated for the descriptive statistics. Data were also compared with norm data by using a one-sample *t*-test. Differences between groups were calculated using Welch’s *t*-test. The effect size was determined using Cohen’s *d.* Moreover, a multiple regression analysis was performed to define the predictors of psychosocial outcomes. An adjusted $R^{2}$ was used as the effect size. Multiple imputation was performed using the Markov chain Monte Carlo (MCMC) approach to counteract possible bias due to missing data. For each variable, the violations of test assumptions, such as normality and homogeneity of variance, were checked. The level of significance was set at p<0.05, two-sided. The statistical analyses were performed using SPSS Statistics 26 (IBM, Armonk, NY, USA).

## 3. Results

### 3.1. Characterstics of the Study Population

**Figure 1:**
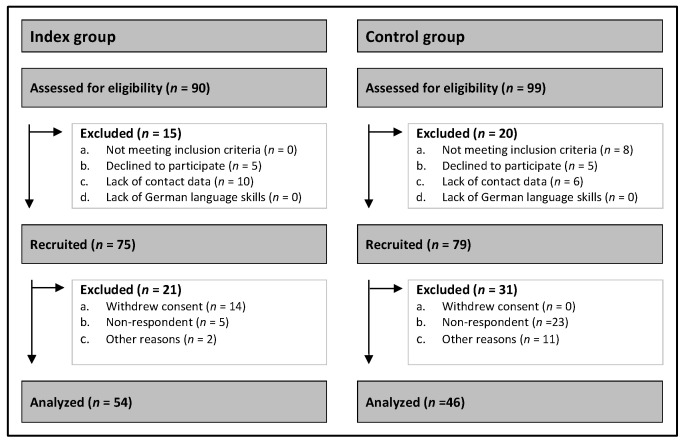
CONSORT flow diagram.

As shown in Figure 1, 189 children were eligible and finally 100 children were included into the study. Table 1 shows the sociodemographic and disease characteristics of the participating families in the index and control groups. Overall, the index and control groups had similar demographics. No relevant difference was found regarding the children’s age between the index and control groups. Moreover, there was only a difference due to the gender distribution within the index group, which had more girls than boys, and the control group had more boys than girls. Similarly, no significant differences were found regarding the parents’ gender, marital status, employment, and education level. Nevertheless, there was a significant difference between the fathers’ age in the index and the control group. The fathers in the control group were older than those in the index group.

Neuroblastoma tumor entity was the most common, followed by nephroblastoma and hepatoblastoma. Rhabdoid tumors were the least represented. Within the control group, bone fracture was the most frequent cause of treatment, followed by other causes, such as phimosis and hernia. 

### 3.2. Differences in Psychosocial Variables between Children with Embryonal Abdominal Tumors, Norm Data, and the Control Group

Table 2 shows the distribution of the overall parent-reported HRQoL and mental health of the child as well as the subscales in the index group and the control group from the perspective of both mothers and fathers. Moreover, a comparison to the norm data is provided. 

In the index group, parent-reported overall HRQoL in both parent perspectives were significantly lower compared to the norm data. Regarding the HRQoL subscales, significant differences were found for physical functionality and emotional and social functionality between the index group and the norm data in the maternal assessment. In the paternal assessment, a significant difference was only found for emotional functionality. The effect sizes varied from low to medium.

Overall, the parent-reported mental health of children in the index group was also significantly decreased compared to the norm data. This applied to both the maternal and the paternal assessment. Regarding the mental health subscales, mothers in the index group reported more conduct and peer problems than the norm data. Additionally, mothers and fathers in the index group reported more hyperactivity than the norm data. 

Moreover, the overall parent-reported HRQoL and mental health scores were not significantly different in the index group compared to the control group. However, the *p*-value was under the threshold for significance in the HRQoL subscale of social functionality, with the maternal and paternal assessments of the index group showing lower values than those of the control group. All other HRQoL and mental health subscales did not reach significance.

### 3.3. Predictors for the Psychosocial Outcomes of Children with Embryonal Abdominal Tumors

Table 3 shows multiple regression models with age, gender, and level of care as three disease-unspecific predictors of psychosocial outcomes for children with embryonal abdominal tumors. The analysis showed that having a level of care was significantly associated with a decreased maternal-reported HRQoL of the affected child. Moreover, the female gender was also associated with a decreased maternal-reported HRQoL and a higher age. The overall model was significant and could explain 19% of the variance in the maternal-reported HRQoL. Regarding maternal-reported mental health, no significant predictor could be identified. The overall model could explain 7% of the variance in maternal-reported mental health, without reaching significance.

Moreover, regarding paternal-reported HRQoL, no significant predictor could be identified. The overall model could explain 14% of the variance in the maternal-reported mental health without reaching significance. Regarding paternal-reported mental health, having a level of care was significantly associated with a worse mental health of the children. The overall model was found to be significant and could explain 53% of the variance.

## 4. Discussion

Childhood cancer is associated with major challenges in everyday life [3,36]. Lifelong physical limitations and lengthy treatments represent a high stress level for the children and their families [12]. While somatic treatment has been increasingly optimized [18,19], the psychosocial situation of children with an embryonal abdominal tumor has been little studied. The present study showed that children with embryonal abdominal tumors had significantly worse paternal- as well as maternal-reported HRQoL and mental health compared to the norm data. In addition, mothers rated their children’s health-related quality of life as well as mental health worse than fathers, especially in terms of physical and school functionality as well as conduct and peer problems. Gender-specific differences between mothers and fathers regarding the assessment of their children’s psychosocial situation are a common phenomenon and known from the previous literature [37]. This suggests that there is still a gap in health care regarding the psychosocial situation of children with embryonal abdominal tumors.

However, contrary to expectations, the HRQoL and mental health of children with embryonal abdominal tumors were not significantly worse than that of children in the control group. These findings differ from those of other studies [25,26,27,28,29,30,31]. An exception is social aspects, in which our study population also differs significantly from the control group. A previous study has shown that contact with hospitals in childhood is fundamentally associated with a high level of stress for the entire family and leads to decreased HRQoL and mental health [38]. This could explain our results, considering that our control group was not fully healthy at the time of the survey but also received surgery. Therefore, it can be assumed that the diathesis–stress model can be used for children and their families who come into contact with the hospital, especially for children with embryonal abdominal tumors.

On the other hand, the results may indicate that the staff’s comprehensive and competent care may positively impact HRQoL and mental health. Moreover, the diagnostic [16,17] as well as the therapy options have also been continuously developed and improved [18,19], which has led to a significant increase in life expectancy [13,14,15]. This good prognosis of the disease can also be used to improve the HRQoL.

Nevertheless, one particular exception was the social domains, in which the children with embryonal abdominal tumors performed significantly worse than the children in the control group. These findings are consistent with the current literature [25,26,31] and suggest that coping with daily challenges, such as role finding within the peer group and integration into a fixed social environment, is especially difficult for children with embryonal abdominal tumors due to the many additional physical as well as emotional challenges, as it has been shown for rare diseases in other studies [30]. Furthermore, treating embryonal abdominal tumors is associated with many hospitalizations [9], leading to incoherent contact with the peer group. In contrast, the limitations experienced by the control group due to outpatient surgery were time-limited. Development in the social environment was thus only impaired for a short time. This finding can be supported by the fact that, compared to the control group, the index group suffers from much more extensive physical damage from the treatment, which requires an elaborate long-term treatment and increasingly impairs the development of social aspects [39]. These findings may indicate a possible gap in care due to children with embryonal abdominal tumors [1,2,3].

Our final objective was to determine the predictors of the respective psychosocial outcome variables of children with embryonal abdominal tumors. Level of care was a significant predictor for the maternal-reported HRQoL. These results align with those of previous studies showing that a higher physical burden is associated with a decreased HRQoL [39]. The physical burden could lead to a limited participation in out-of-hospital activities and degrade social functionality. In the future, this could indicate that more attention should be paid to children with a level of care, especially regarding their social development. For example, playgrounds and play spaces could be made more accessible for children with special needs, as well as schoolyards and kindergarten buildings. Moreover, having a level of care also seems to be a significant predictor of paternal-reported mental health. However, the results must be treated cautiously, as significance is likely due to the low case number of fathers. Nevertheless, our analysis may provide evidence that having a level of care may be a risk factor for reduced HRQoL and mental health. In contrast, the child’s age seems to play only a secondary role.

## 5. Study Limitations

The present study is accompanied by limitations: (1) The HRQoL and mental health of the children were exclusively ascertained by third-party assessment of the parents. Parental assessment may be biased by stress on the parents themselves. (2) We cannot exclude the possibility of a non-response bias. It can be assumed that the children of the participating families generally have a better HRQoL and mental health than those whose families did not respond. (3) Although we included all four entities of embryonal abdominal tumor, there were more children with neuroblastoma and nephroblastoma. Thus, a larger study population should be recruited for a more comprehensive assessment of all tumor entities, such as neuroblastoma, nephroblastoma, hepatoblastoma, and rhabdoid tumors. (4) Due to the rarity of the disease and the exclusive survey at one study center, the study population was correspondingly small. For further analyses, the disease should be considered across cities in Germany. (5) In addition to level of care, other predictors, such as time since diagnosis, may have played an important role in the HRQoL and mental health of affected children. Future studies should consider the already identified important disease-specific and psychosocial factors [37]. (6) Because we have a treatment period of 2012–2022, there may be differences between groups regarding diagnostic and treatment. This could also influence the variability in the children’s HRQoL and mental health. (7) All families were recruited in northern Germany. Thus, a transfer to other countries should be performed with caution.

## 6. Conclusions

Embryonal abdominal tumors affect the well-being of children. Affected patients have significantly lower HRQoL and mental health values than the norm data. Our study provided additional evidence of a still-existing psychosocial care gap for children with embryonal abdominal tumors and highlights the specific psychosocial impairment of the affected children, especially with regard to social development.

## Figures and Tables

**Table 1:** Sociodemographic and disease characteristics of the index and control groups.

	Index Group (*n* = 54 Families)		Control Group (*n* = 46 Families)		Group Differences (*p*-Value)
Characteristics	*M*	*SD*	*M*	*SD*	
Patient’s age (years)	8.33	3.3	9.51	4.48	0.201
Mother’s age (years)	41.39	4.95	40.28	6.78	0.444
Father’s age (years)	42.81	3.2	47.13	6.79	**0.021**
Number of children (mothers)	2.13	1.18	2.07	0.75	0.795
Number of children (fathers)	2.14	1.46	2.24	1.02	0.775
Time since initial diagnosis (years)	5.50	2.54	*-*	-	-
Time since first surgery (years)	5.37	2.54	*-*	-	-
**Parents**	*n*	%	*n*	%	
Parent’s gender (mothers/fathers)	48/19	88.9/35.2	41/24	89.1/52.2	0.970/0.090
Marital status (mothers/fathers)					0.737/0.478
Married/Living together	40/20	83.3/91.0	34/26	79.0/89.6	
Single	6/1	12.5/4.5	6/1	14.0/3.4	
Divorced	2/1	4.2/4.5	3/1	7.0/3.4	
Not stated		0.0/0.0	0/1	0.0/3.4	
Education (mothers/ fathers)					0.712/0.942
Lower-middle education	17/9	35.4/40.9	15/11	34.9/37.9	
Higher education	29/12	60.4/54.6	26/16	60.5/55.2	
Not stated	2/1	4.2/4.5	2/2	4.6/6.9	
Employment ^1^ (mothers/fathers)					0.139/0.666
Fully employed	7/17	14.6/77.3	14/25	32.6/86.2	
Partly employed	29/5	60.4/22.7	22/3	51.2/10.3	
No employment	4/0	8.3/0.0	6/1	13.9/3.5	
Not stated	8/0	16.7/0.0	1/0	2.3/0.0	
**Patients**	*n*	%	*n*	%	
Patient’s gender					**0.007**
Female	32	59.3	15	32.6	
Male	22	40.7	31	67.4	
Patient receives level of care ^2^					
Yes	22	46.3	-	-	
No No data	25 7	40.7 13.0	-	-	
Patient solid abdominal tumors					
Neuroblastoma	24	44.4	-	-	
Nephroblastoma	22	40.7	-	-	
Hepatoblastoma	6	11.1	-	-	
Rhabdoid Tumor	2	3.7	-	-	
Others	0	0	-	-	
Children control group deseases					
Hernia	-	-	3	6.5	
Fimosis	-	-	2	4.3	
Median Neck Cyst	-	-	0	0	
Bone Fracture	-	-	21	45.7	
Testicular Malposition	-	-	0	0	
Others	-	-	20	43.5	

Note: ^1^ Refers to the last 12 months. ^2^ Refers to the decision for the classification in the care insurance according to German long-term care insurance.

**Table 2:** Distribution of parent-reported Health-Related Quality of Life (HRQoL) and mental health for the index group, the control group, and the norm data of PedsQL and SDQ.

	Index Group (a)		Control Group (b)		Norm Data (c)		Differences a vs. b	Effect Size a vs. b	Differences a vs. c	Effect Size a vs. c
	*M*	*SD*	*M*	*SD*	*M*	*SD*	*p*	*d*	*p*	*d*
**Mothers (*n* = 48)**										
Children’s HRQoL total score (PedsQL)	79.03	15.81	85	13.91	86.1	11.2	0.052	−0.415	**0.003**	−0.448
(1) Physical functionality	86.15	19.08	89.63	16.26	91.7	10.7	0.272	−0.231	**0.049**	−0.291
(2) Emotional functionality	70.31	21.48	76.68	21.33	81.2	17.0	0.166	−0.297	**<0.001**	−0.507
(3) Social functionality	79.17	22.01	90.65	12.39	86.9	16.08	**0.003**	−0.630	**0.019**	−0.351
(4) School functionality	78.9	22.38	82.29	18.51	82.1	19.6	0.441	−0.164	0.332	−0.143
Children’s mental health total score (SDQ)	12.17	4.15	11.10	3.75	8.5	7.22	0.202	0.270	**<0.001**	0.883
(1) Emotional symptoms	2.54	2.42	1.69	2.02	2.0	2.41	0.073	0.379	0.128	0.224
(2) Conduct problems	2.63	1.08	2.71	1.24	2.2	2.41	0.718	−0.77	**0.009**	0.393
(3) Hyperactivity	4.94	1.58	5.33	1.48	3.2	3.61	0.222	−0.259	**<0.001**	1.102
(4) Peer problems	2.04	2.16	1.36	2.08	1.4	2.41	0.119	0.332	**0.039**	0.307
(5) Prosocial behavior	8	1.96	8.05	1.90	8.3	2.41	0.907	−0.025	0.294	−0.153
**Fathers (*n* = 19)**										
Children’s HRQoL total score (PedsQL)	78.36	11.84	82,99	13.01	86.1	11.2	0.161	−0.439	**0.011**	−0.654
(1) Physical functionality	83,95	18.23	86.67	18.98	91.7	10.7	0.519	−0.201	0.080	−0.425
(2) Emotional functionality	69.19	19.3	72.66	19.14	81.2	17.0	0.432	−0.247	**0.014**	−0.622
(3) Social functionality	82.24	12.72	91.67	12.77	86.9	16.8	**0.021**	−0.740	0.127	−0.367
(4) School functionality	76.39	18.58	81.94	19.45	82.1	19.6	0.353	−0.291	0.210	−0.307
Children’s mental health total score (SDQ)	11.18	3.49	10.43	2.92	8.5	7.22	0.482	0.234	**0.006**	0.768
(1) Emotional symptoms	2.28	2.49	1.35	1.37	2.0	2.41	0.167	0.479	0.642	0.111
(2) Conduct problems	2.35	0.93	2.74	1.29	2.2	2.41	0.278	−0.336	0.508	0.164
(3) Hyperactivity	4.53	1.51	5.30	1.33	3.2	3.61	0.101	−0.551	**0.002**	0.883
(4) Peer problems	1.88	1.69	1.04	1.64	1.4	2.41	0.125	0.505	0.257	0.285
(5) Prosocial behavior	7.71	1.61	7.88	1.83	8.3	2.41	0.756	−0.097	0.148	−0.369

Note: HRQoL = Health-Related Quality of Life. SF15 = Pediatric Quality of Life Inventory Short Form 15. SDQ = Strength and Difficulties Questionnaire. Comparison between groups was assessed with a one-sample *t*-test and Welch’s *t*-test. Effect size was calculated using Cohen’s *d*. The norm data PedsQL of healthy children and adolescents, according to the parent report [33]. The norm Data SDQ of healthy children and adolescents, according to the parent report [35].

**Table 3:** Prediction of psychosocial measures of children with embryonal abdominal tumors.

	Constant			Age			Gender			Level of Care			Overall Model	
	*b*	95% CI	*p*	*b*	95% CI	*p*	*b*	95% CI	*p*	*b*	95% CI	*p*	R^2^	*p*
Mothers														
Children’s HRQoL	**90.07**	**[75.79, 104.36]**	**<0.001**	−0.01	[−1.46, 1.43]	0.988	−7.20	[−5.79, 8.96]	0.115	**−14.08**	**[−23.01, −5.14]**	**0.003**	**0.19**	**0.007**
Children’s mental health	**10.30**	**[6.22, 14.38]**	**<0.001**	−0.03	[−0.44, −0.23]	0.885	2.21	[−0.37, 4.79]	0.091	1.61	[−0.94, 4.16]	0.211	0.04	0.198
Fathers														
Children’s HRQoL	**74.99**	**[56.03, 93.95]**	**<0.001**	1.14	[−1.01, 3.30]	0.276	−5.68	[−16.93, 5.58]	0.299	−8.73	[−21.08, 3.62]	0.153	0.14	0.162
Children’s mental health	**11.11**	**[6.74, 15.48]**	**<0.001**	−0.24	[−0.74, 0.26]	0.321	1.34	[−1.30, 3.97]	0.293	**5.76**	**[2.74, 8.77]**	**0.001**	**0.53**	**0.005**

Note: CI = Confidence interval. HRQoL = Health-Related Quality of Life. Gender of child: female = 1, male = 0. Level of care: yes = 1, no = 0.

## Data Availability

The data presented in this study are available upon request from the corresponding author. The data are not publicly available due to restrictions, e.g., privacy.
